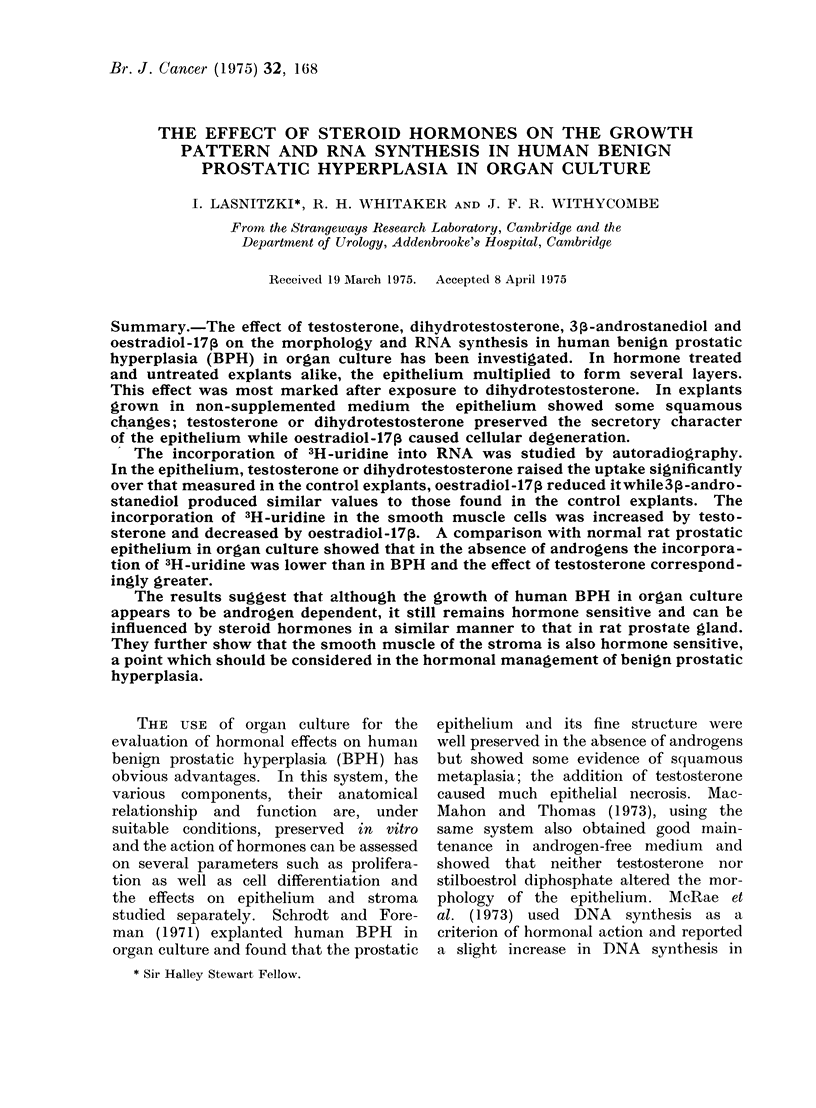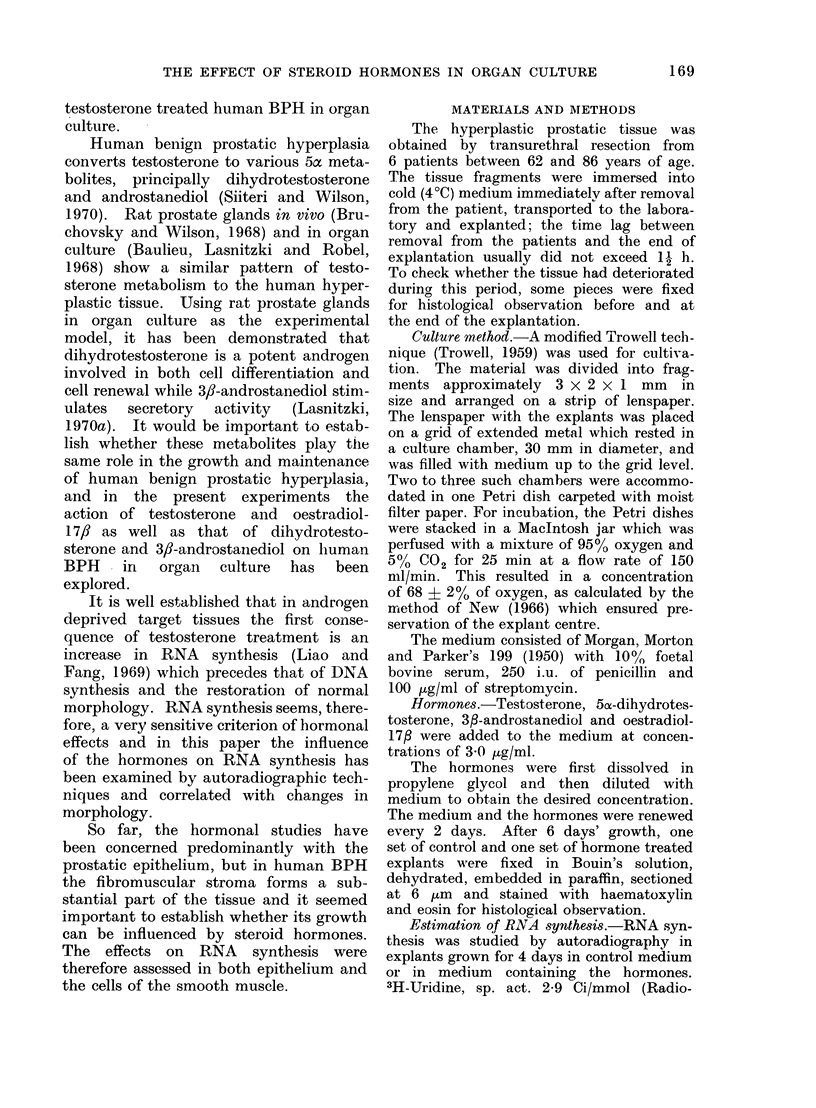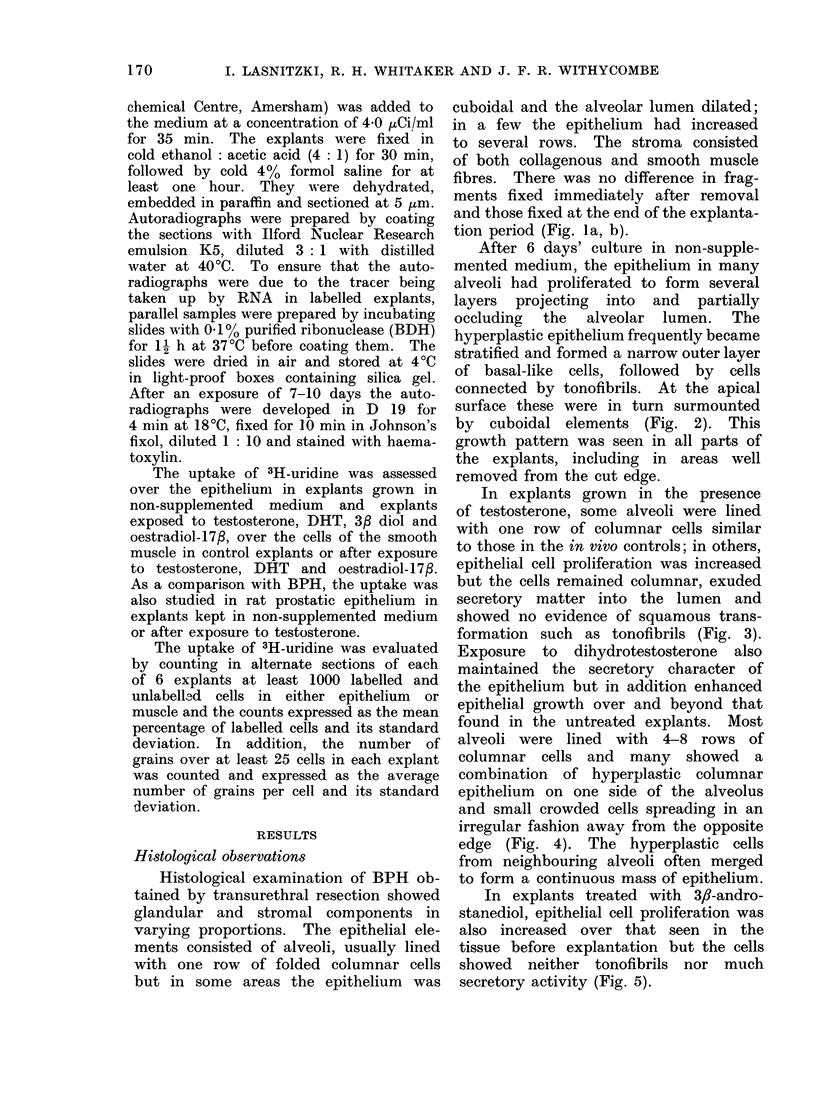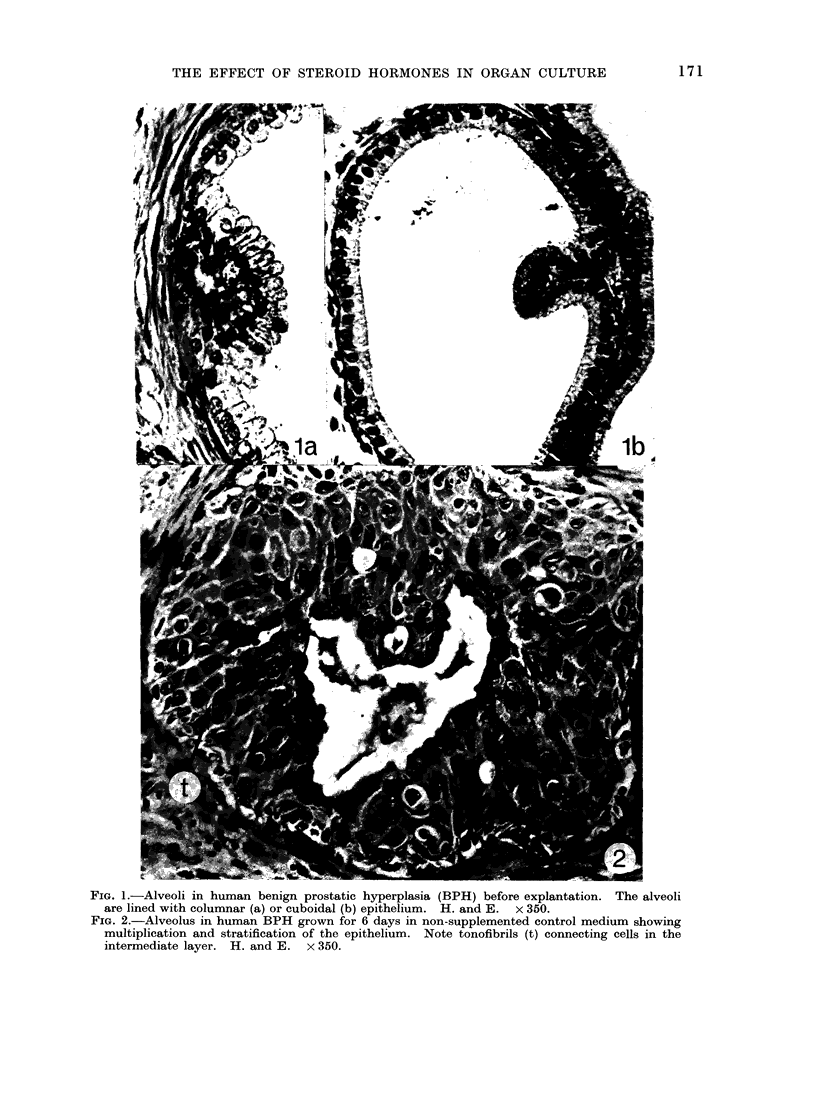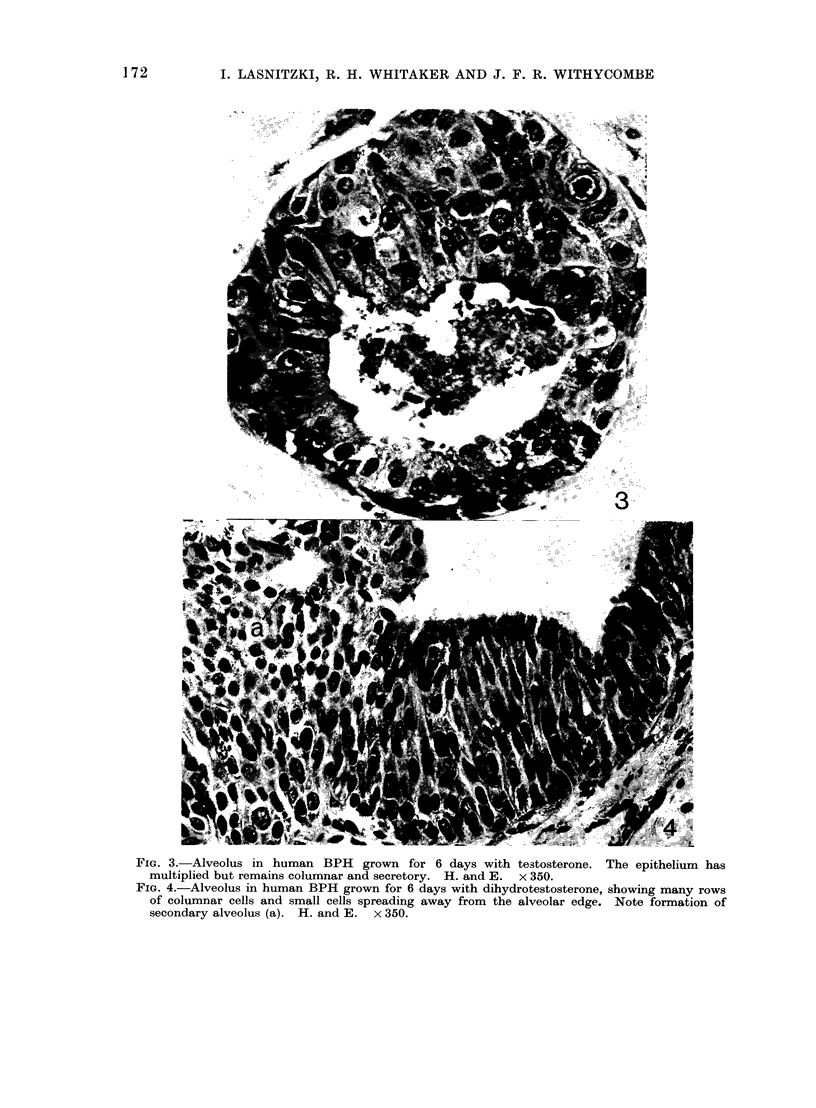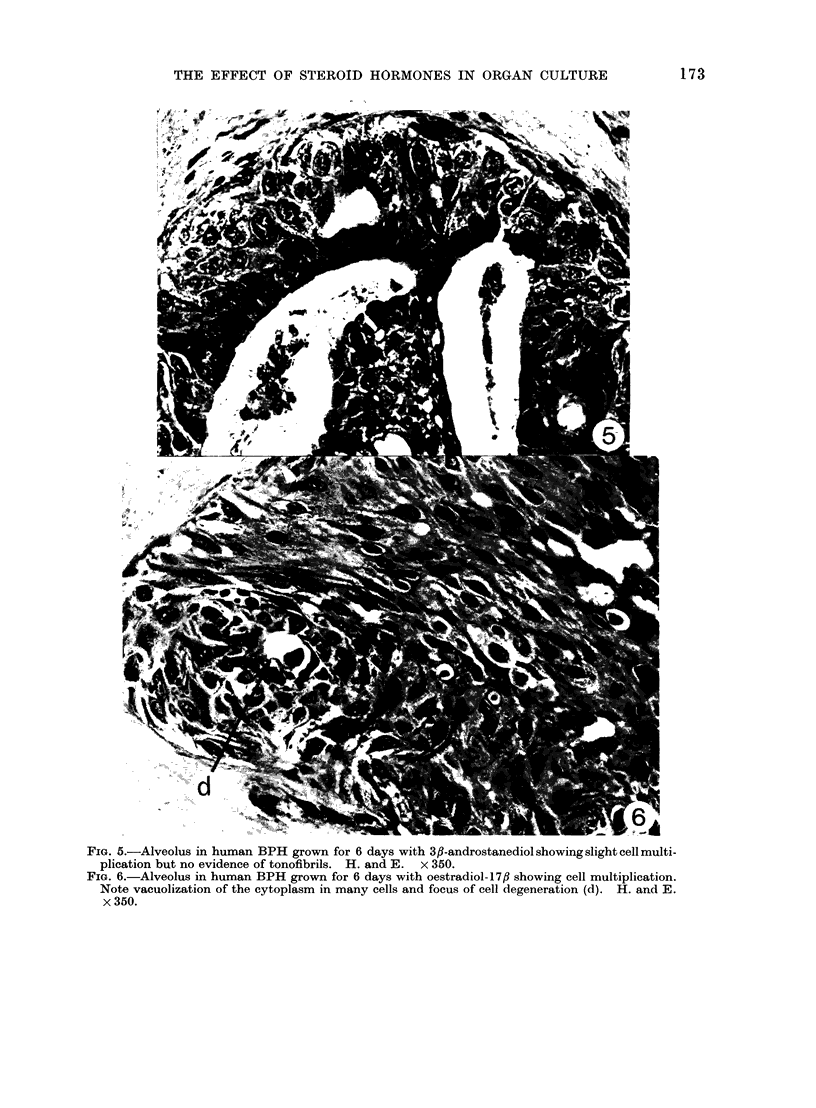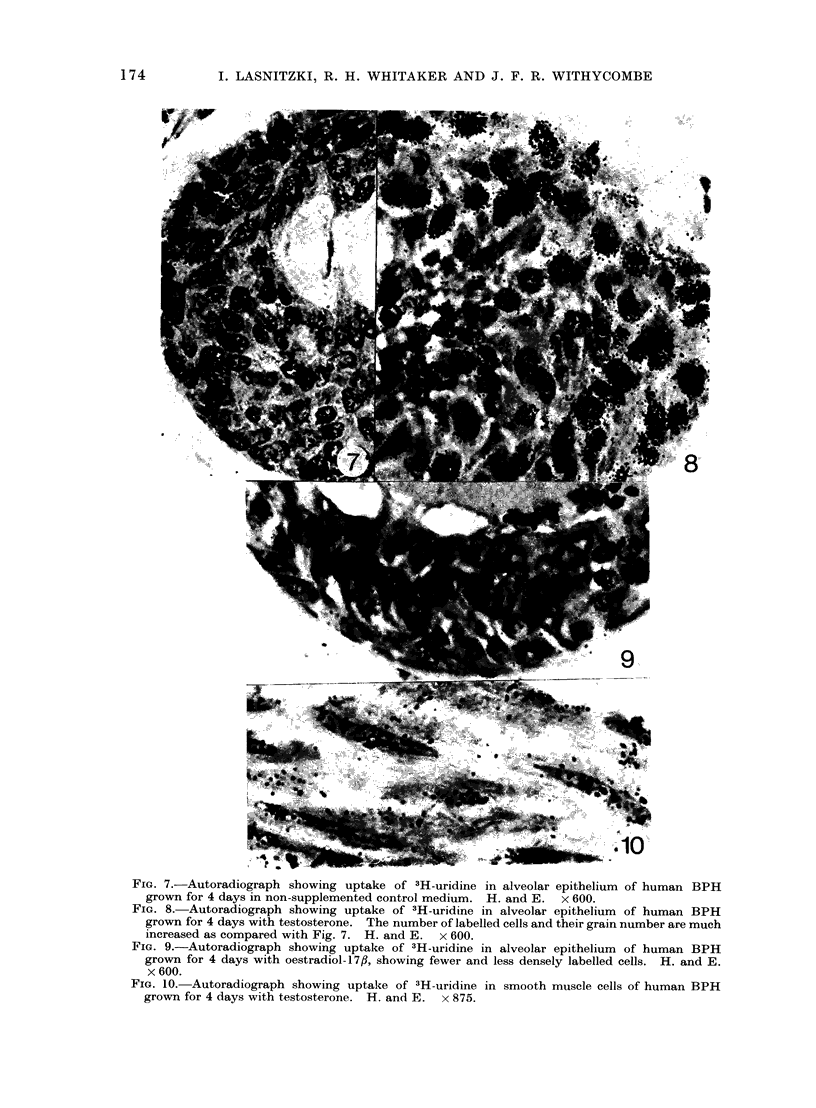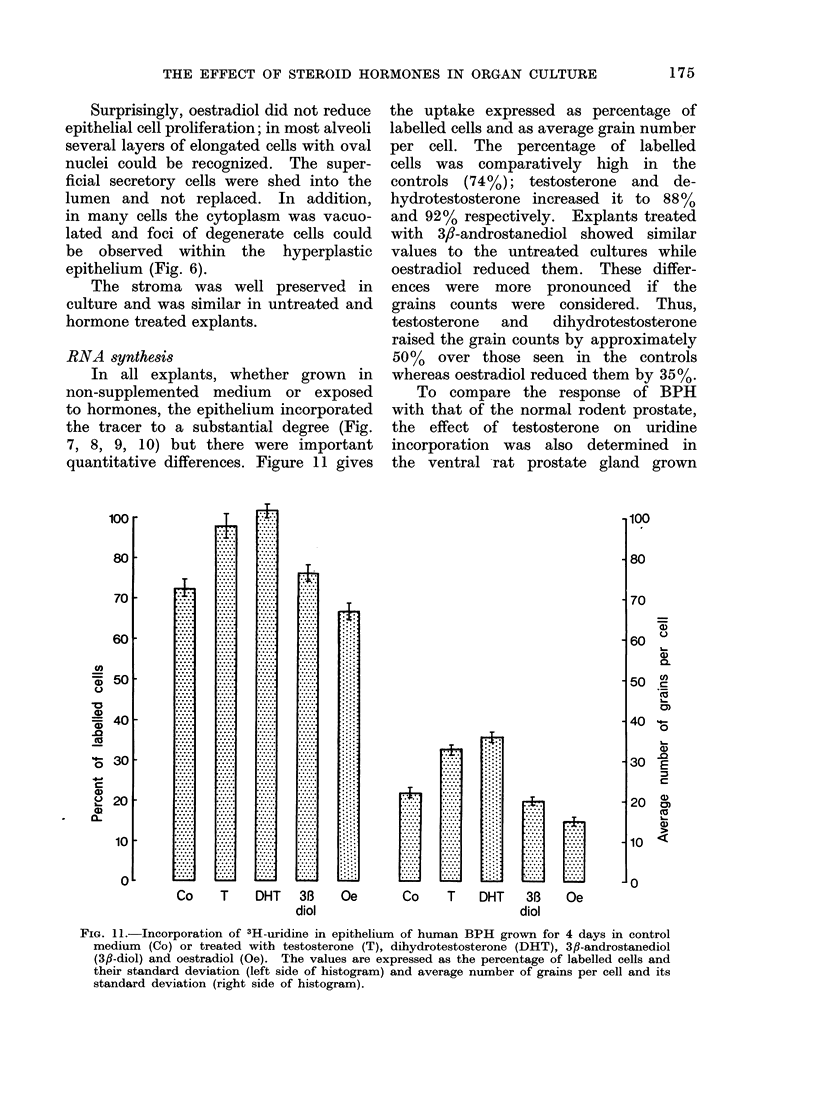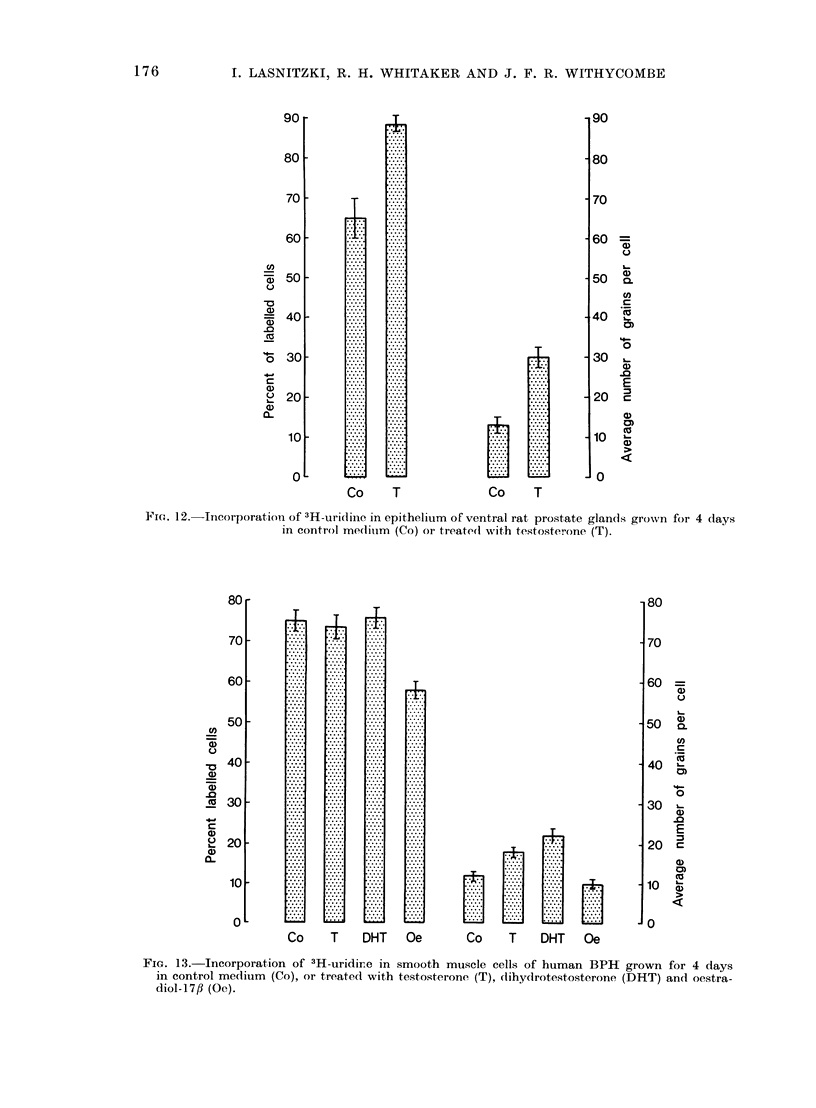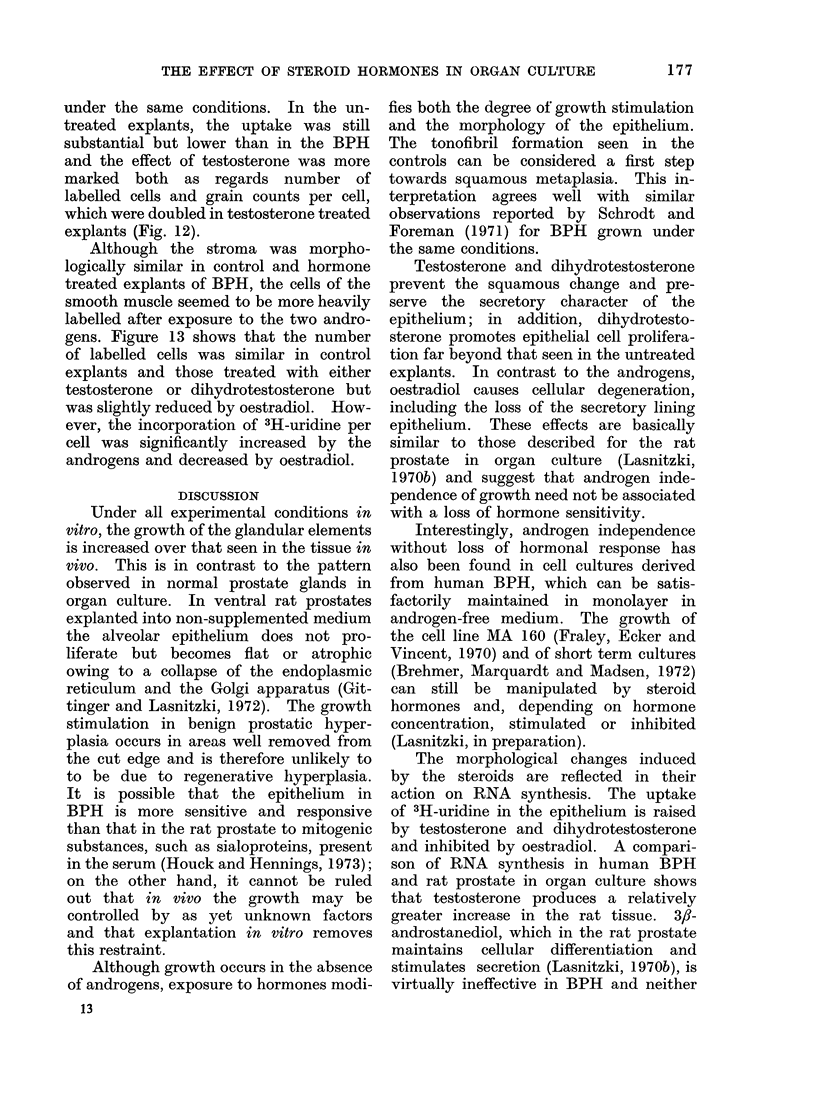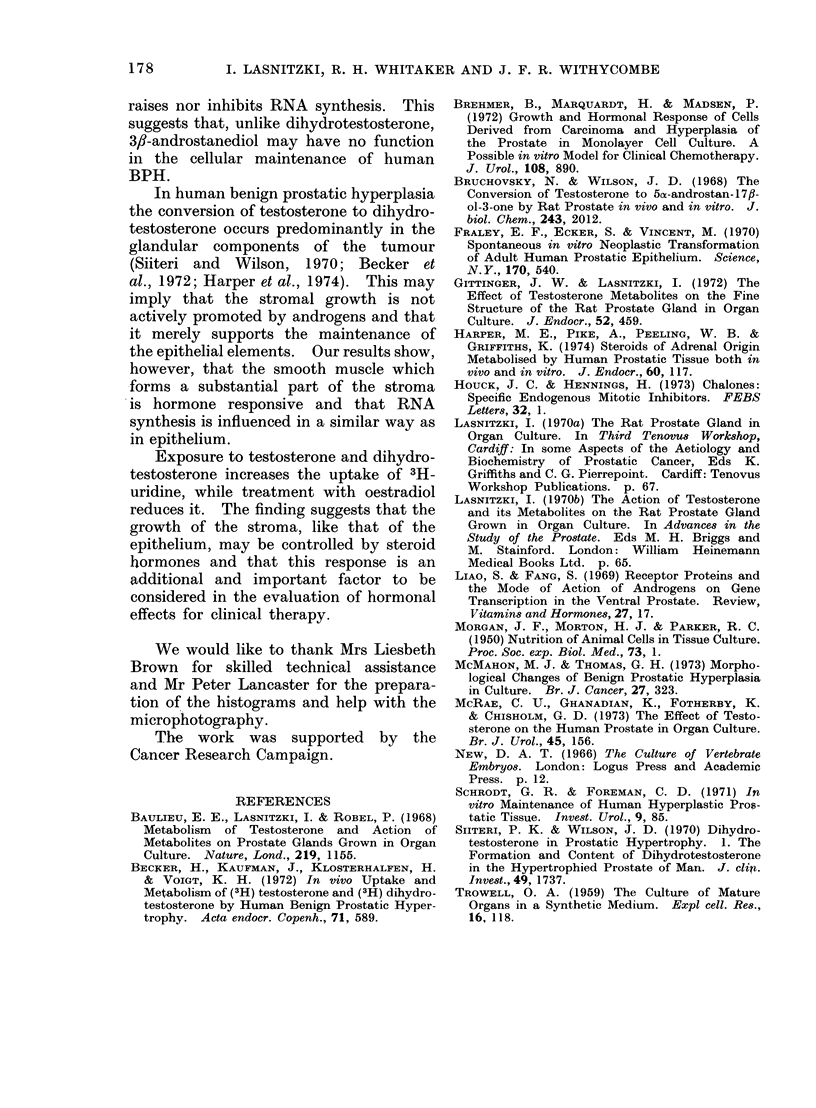# The effect of steroid hormones on the growth pattern and RNA synthesis in human benign prostatic hyperplasia in organ culture.

**DOI:** 10.1038/bjc.1975.146

**Published:** 1975-08

**Authors:** I. Lasnitzki, R. H. Whitaker, J. F. Withycombe

## Abstract

**Images:**


*Br. J. Cancer* (1975) **32**, 168

### THE EFFECT OF STEROID HORMONES ON THE GROWTH PATTERN AND RNA SYNTHESIS IN HUMAN BENIGN PROSTATIC HYPERPLASIA IN ORGAN CULTURE

I. LASNITZKI*, R. H. WHITAKER AND J. F. R. WITHYCOMBE

*From the Strangeways Research Laboratory, Cambridge and the Department of Urology, Addenbrooke's Hospital, Cambridge*

Received 19 March 1975. Accepted 8 April 1975

**Summary.—The effect of testosterone, dihydrotestosterone, 3β-androstanediol and oestradiol-17β on the morphology and RNA synthesis in human benign prostatic hyperplasia (BPH) in organ culture has been investigated. In hormone treated and untreated explants alike, the epithelium multiplied to form several layers. This effect was most marked after exposure to dihydrotestosterone. In explants grown in non-supplemented medium the epithelium showed some squamous changes; testosterone or dihydrotestosterone preserved the secretory character of the epithelium while oestradiol-17β caused cellular degeneration.**

**The incorporation of $^3$H-uridine into RNA was studied by autoradiography. In the epithelium, testosterone or dihydrotestosterone raised the uptake significantly over that measured in the control explants, oestradiol-17β reduced it while 3β-androstanediol produced similar values to those found in the control explants. The incorporation of $^3$H-uridine in the smooth muscle cells was increased by testosterone and decreased by oestradiol-17β. A comparison with normal rat prostatic epithelium in organ culture showed that in the absence of androgens the incorporation of $^3$H-uridine was lower than in BPH and the effect of testosterone correspondingly greater.**

**The results suggest that although the growth of human BPH in organ culture appears to be androgen dependent, it still remains hormone sensitive and can be influenced by steroid hormones in a similar manner to that in rat prostate gland. They further show that the smooth muscle of the stroma is also hormone sensitive, a point which should be considered in the hormonal management of benign prostatic hyperplasia.**

THE USE of organ culture for the evaluation of hormonal effects on human benign prostatic hyperplasia (BPH) has obvious advantages. In this system, the various components, their anatomical relationship and function are, under suitable conditions, preserved *in vitro* and the action of hormones can be assessed on several parameters such as proliferation as well as cell differentiation and the effects on epithelium and stroma studied separately. Schrodt and Foreman (1971) explanted human BPH in organ culture and found that the prostatic epithelium and its fine structure were well preserved in the absence of androgens but showed some evidence of squamous metaplasia; the addition of testosterone caused much epithelial necrosis. MacMahon and Thomas (1973), using the same system also obtained good maintenance in androgen-free medium and showed that neither testosterone nor stilboestrol diphosphate altered the morphology of the epithelium. McRae *et al.* (1973) used DNA synthesis as a criterion of hormonal action and reported a slight increase in DNA synthesis in

* Sir Halley Stewart Fellow.

testosterone treated human BPH in organ culture.

Human benign prostatic hyperplasia converts testosterone to various 5α metabolites, principally dihydrotestosterone and androstanediol (Siiteri and Wilson, 1970). Rat prostate glands *in vivo* (Bruchovsky and Wilson, 1968) and in organ culture (Baulieu, Lasnitzki and Robel, 1968) show a similar pattern of testosterone metabolism to the human hyperplastic tissue. Using rat prostate glands in organ culture as the experimental model, it has been demonstrated that dihydrotestosterone is a potent androgen involved in both cell differentiation and cell renewal while 3β-androstanediol stimulates secretory activity (Lasnitzki, 1970*a*). It would be important to establish whether these metabolites play the same role in the growth and maintenance of human benign prostatic hyperplasia, and in the present experiments the action of testosterone and oestradiol-17β as well as that of dihydrotestosterone and 3β-androstanediol on human BPH in organ culture has been explored.

It is well established that in androgen deprived target tissues the first consequence of testosterone treatment is an increase in RNA synthesis (Liao and Fang, 1969) which precedes that of DNA synthesis and the restoration of normal morphology. RNA synthesis seems, therefore, a very sensitive criterion of hormonal effects and in this paper the influence of the hormones on RNA synthesis has been examined by autoradiographic techniques and correlated with changes in morphology.

So far, the hormonal studies have been concerned predominantly with the prostatic epithelium, but in human BPH the fibromuscular stroma forms a substantial part of the tissue and it seemed important to establish whether its growth can be influenced by steroid hormones. The effects on RNA synthesis were therefore assessed in both epithelium and the cells of the smooth muscle.

### MATERIALS AND METHODS

The hyperplastic prostatic tissue was obtained by transurethral resection from 6 patients between 62 and 86 years of age. The tissue fragments were immersed into cold (4 °C) medium immediately after removal from the patient, transported to the laboratory and explanted; the time lag between removal from the patients and the end of explantation usually did not exceed $1\frac{1}{2}$ h. To check whether the tissue had deteriorated during this period, some pieces were fixed for histological observation before and at the end of the explantation.

*Culture method.*—A modified Trowell technique (Trowell, 1959) was used for cultivation. The material was divided into fragments approximately $3 \times 2 \times 1$ mm in size and arranged on a strip of lenspaper. The lenspaper with the explants was placed on a grid of extended metal which rested in a culture chamber, 30 mm in diameter, and was filled with medium up to the grid level. Two to three such chambers were accommodated in one Petri dish carpeted with moist filter paper. For incubation, the Petri dishes were stacked in a MacIntosh jar which was perfused with a mixture of 95% oxygen and 5% $CO_2$ for 25 min at a flow rate of 150 ml/min. This resulted in a concentration of $68 \pm 2\%$ of oxygen, as calculated by the method of New (1966) which ensured preservation of the explant centre.

The medium consisted of Morgan, Morton and Parker's 199 (1950) with 10% foetal bovine serum, 250 i.u. of penicillin and 100 μg/ml of streptomycin.

*Hormones.*—Testosterone, 5α-dihydrotestosterone, 3β-androstanediol and oestradiol-17β were added to the medium at concentrations of 3·0 μg/ml.

The hormones were first dissolved in propylene glycol and then diluted with medium to obtain the desired concentration. The medium and the hormones were renewed every 2 days. After 6 days' growth, one set of control and one set of hormone treated explants were fixed in Bouin's solution, dehydrated, embedded in paraffin, sectioned at 6 μm and stained with haematoxylin and eosin for histological observation.

*Estimation of RNA synthesis.*—RNA synthesis was studied by autoradiography in explants grown for 4 days in control medium or in medium containing the hormones. $^3$H-Uridine, sp. act. 2·9 Ci/mmol (Radiochemical Centre, Amersham) was added to the medium at a concentration of 4·0 $\mu$Ci/ml for 35 min. The explants were fixed in cold ethanol : acetic acid (4 : 1) for 30 min, followed by cold 4% formol saline for at least one hour. They were dehydrated, embedded in paraffin and sectioned at 5 $\mu$m. Autoradiographs were prepared by coating the sections with Ilford Nuclear Research emulsion K5, diluted 3 : 1 with distilled water at 40°C. To ensure that the autoradiographs were due to the tracer being taken up by RNA in labelled explants, parallel samples were prepared by incubating slides with 0·1% purified ribonuclease (BDH) for 1½ h at 37°C before coating them. The slides were dried in air and stored at 4°C in light-proof boxes containing silica gel. After an exposure of 7–10 days the autoradiographs were developed in D 19 for 4 min at 18°C, fixed for 10 min in Johnson's fixol, diluted 1 : 10 and stained with haematoxylin.

The uptake of $^3$H-uridine was assessed over the epithelium in explants grown in non-supplemented medium and explants exposed to testosterone, DHT, 3$\beta$ diol and oestradiol-17$\beta$, over the cells of the smooth muscle in control explants or after exposure to testosterone, DHT and oestradiol-17$\beta$. As a comparison with BPH, the uptake was also studied in rat prostatic epithelium in explants kept in non-supplemented medium or after exposure to testosterone.

The uptake of $^3$H-uridine was evaluated by counting in alternate sections of each of 6 explants at least 1000 labelled and unlabelled cells in either epithelium or muscle and the counts expressed as the mean percentage of labelled cells and its standard deviation. In addition, the number of grains over at least 25 cells in each explant was counted and expressed as the average number of grains per cell and its standard deviation.

### RESULTS

### *Histological observations*

Histological examination of BPH obtained by transurethral resection showed glandular and stromal components in varying proportions. The epithelial elements consisted of alveoli, usually lined with one row of folded columnar cells but in some areas the epithelium was cuboidal and the alveolar lumen dilated; in a few the epithelium had increased to several rows. The stroma consisted of both collagenous and smooth muscle fibres. There was no difference in fragments fixed immediately after removal and those fixed at the end of the explantation period (Fig. 1a, b).

After 6 days' culture in non-supplemented medium, the epithelium in many alveoli had proliferated to form several layers projecting into and partially occluding the alveolar lumen. The hyperplastic epithelium frequently became stratified and formed a narrow outer layer of basal-like cells, followed by cells connected by tonofibrils. At the apical surface these were in turn surmounted by cuboidal elements (Fig. 2). This growth pattern was seen in all parts of the explants, including in areas well removed from the cut edge.

In explants grown in the presence of testosterone, some alveoli were lined with one row of columnar cells similar to those in the *in vivo* controls; in others, epithelial cell proliferation was increased but the cells remained columnar, exuded secretory matter into the lumen and showed no evidence of squamous transformation such as tonofibrils (Fig. 3). Exposure to dihydrotestosterone also maintained the secretory character of the epithelium but in addition enhanced epithelial growth over and beyond that found in the untreated explants. Most alveoli were lined with 4–8 rows of columnar cells and many showed a combination of hyperplastic columnar epithelium on one side of the alveolus and small crowded cells spreading in an irregular fashion away from the opposite edge (Fig. 4). The hyperplastic cells from neighbouring alveoli often merged to form a continuous mass of epithelium.

In explants treated with 3$\beta$-androstanediol, epithelial cell proliferation was also increased over that seen in the tissue before explantation but the cells showed neither tonofibrils nor much secretory activity (Fig. 5).

FIG. 1.—Alveoli in human benign prostatic hyperplasia (BPH) before explantation. The alveoli are lined with columnar (a) or cuboidal (b) epithelium. H. and E. ×350.

FIG. 2.—Alveolus in human BPH grown for 6 days in non-supplemented control medium showing multiplication and stratification of the epithelium. Note tonofibrils (t) connecting cells in the intermediate layer. H. and E. ×350.

FIG. 3.—Alveolus in human BPH grown for 6 days with testosterone. The epithelium has multiplied but remains columnar and secretory. H. and E. ×350.

FIG. 4.—Alveolus in human BPH grown for 6 days with dihydrotestosterone, showing many rows of columnar cells and small cells spreading away from the alveolar edge. Note formation of secondary alveolus (a). H. and E. ×350.

FIG. 5.—Alveolus in human BPH grown for 6 days with 3$\beta$-androstanediol showing slight cell multiplication but no evidence of tonofibrils. H. and E. $\times 350$.

FIG. 6.—Alveolus in human BPH grown for 6 days with oestradiol-17$\beta$ showing cell multiplication. Note vacuolization of the cytoplasm in many cells and focus of cell degeneration (d). H. and E. $\times 350$.

FIG. 7.—Autoradiograph showing uptake of $^{3}$H-uridine in alveolar epithelium of human BPH grown for 4 days in non-supplemented control medium. H. and E. $\times 600$.

FIG. 8.—Autoradiograph showing uptake of $^{3}$H-uridine in alveolar epithelium of human BPH grown for 4 days with testosterone. The number of labelled cells and their grain number are much increased as compared with Fig. 7. H. and E. $\times 600$.

FIG. 9.—Autoradiograph showing uptake of $^{3}$H-uridine in alveolar epithelium of human BPH grown for 4 days with oestradiol-17$\beta$, showing fewer and less densely labelled cells. H. and E. $\times 600$.

FIG. 10.—Autoradiograph showing uptake of $^{3}$H-uridine in smooth muscle cells of human BPH grown for 4 days with testosterone. H. and E. $\times 875$.

Surprisingly, oestradiol did not reduce epithelial cell proliferation; in most alveoli several layers of elongated cells with oval nuclei could be recognized. The superficial secretory cells were shed into the lumen and not replaced. In addition, in many cells the cytoplasm was vacuolated and foci of degenerate cells could be observed within the hyperplastic epithelium (Fig. 6).

The stroma was well preserved in culture and was similar in untreated and hormone treated explants.

*RNA synthesis*

In all explants, whether grown in non-supplemented medium or exposed to hormones, the epithelium incorporated the tracer to a substantial degree (Fig. 7, 8, 9, 10) but there were important quantitative differences. Figure 11 gives the uptake expressed as percentage of labelled cells and as average grain number per cell. The percentage of labelled cells was comparatively high in the controls (74%); testosterone and dehydrotestosterone increased it to 88% and 92% respectively. Explants treated with 3$\beta$-androstanediol showed similar values to the untreated cultures while oestradiol reduced them. These differences were more pronounced if the grains counts were considered. Thus, testosterone and dihydrotestosterone raised the grain counts by approximately 50% over those seen in the controls whereas oestradiol reduced them by 35%.

To compare the response of BPH with that of the normal rodent prostate, the effect of testosterone on uridine incorporation was also determined in the ventral rat prostate gland grown

FIG. 11.—Incorporation of $^3$H-uridine in epithelium of human BPH grown for 4 days in control medium (Co) or treated with testosterone (T), dihydrotestosterone (DHT), 3$\beta$-androstanediol (3$\beta$-diol) and oestradiol (Oe). The values are expressed as the percentage of labelled cells and their standard deviation (left side of histogram) and average number of grains per cell and its standard deviation (right side of histogram).

FIG. 12.—Incorporation of $^{3}H$-uridine in epithelium of ventral rat prostate glands grown for 4 days in control medium (Co) or treated with testosterone (T).

FIG. 13.—Incorporation of $^{3}H$-uridine in smooth muscle cells of human BPH grown for 4 days in control medium (Co), or treated with testosterone (T), dihydrotestosterone (DHT) and oestradiol-17$\beta$ (Oe).

under the same conditions. In the untreated explants, the uptake was still substantial but lower than in the BPH and the effect of testosterone was more marked both as regards number of labelled cells and grain counts per cell, which were doubled in testosterone treated explants (Fig. 12).

Although the stroma was morphologically similar in control and hormone treated explants of BPH, the cells of the smooth muscle seemed to be more heavily labelled after exposure to the two androgens. Figure 13 shows that the number of labelled cells was similar in control explants and those treated with either testosterone or dihydrotestosterone but was slightly reduced by oestradiol. However, the incorporation of $^{3}$H-uridine per cell was significantly increased by the androgens and decreased by oestradiol.

### DISCUSSION

Under all experimental conditions *in vitro*, the growth of the glandular elements is increased over that seen in the tissue *in vivo*. This is in contrast to the pattern observed in normal prostate glands in organ culture. In ventral rat prostates explanted into non-supplemented medium the alveolar epithelium does not proliferate but becomes flat or atrophic owing to a collapse of the endoplasmic reticulum and the Golgi apparatus (Gittinger and Lasnitzki, 1972). The growth stimulation in benign prostatic hyperplasia occurs in areas well removed from the cut edge and is therefore unlikely to to be due to regenerative hyperplasia. It is possible that the epithelium in BPH is more sensitive and responsive than that in the rat prostate to mitogenic substances, such as sialoproteins, present in the serum (Houck and Hennings, 1973); on the other hand, it cannot be ruled out that *in vivo* the growth may be controlled by as yet unknown factors and that explantation *in vitro* removes this restraint.

Although growth occurs in the absence of androgens, exposure to hormones modifies both the degree of growth stimulation and the morphology of the epithelium. The tonofibril formation seen in the controls can be considered a first step towards squamous metaplasia. This interpretation agrees well with similar observations reported by Schrodt and Foreman (1971) for BPH grown under the same conditions.

Testosterone and dihydrotestosterone prevent the squamous change and preserve the secretory character of the epithelium; in addition, dihydrotestosterone promotes epithelial cell proliferation far beyond that seen in the untreated explants. In contrast to the androgens, oestradiol causes cellular degeneration, including the loss of the secretory lining epithelium. These effects are basically similar to those described for the rat prostate in organ culture (Lasnitzki, 1970*b*) and suggest that androgen independence of growth need not be associated with a loss of hormone sensitivity.

Interestingly, androgen independence without loss of hormonal response has also been found in cell cultures derived from human BPH, which can be satisfactorily maintained in monolayer in androgen-free medium. The growth of the cell line MA 160 (Fraley, Ecker and Vincent, 1970) and of short term cultures (Brehmer, Marquardt and Madsen, 1972) can still be manipulated by steroid hormones and, depending on hormone concentration, stimulated or inhibited (Lasnitzki, in preparation).

The morphological changes induced by the steroids are reflected in their action on RNA synthesis. The uptake of $^{3}$H-uridine in the epithelium is raised by testosterone and dihydrotestosterone and inhibited by oestradiol. A comparison of RNA synthesis in human BPH and rat prostate in organ culture shows that testosterone produces a relatively greater increase in the rat tissue. $3\beta$-androstanediol, which in the rat prostate maintains cellular differentiation and stimulates secretion (Lasnitzki, 1970*b*), is virtually ineffective in BPH and neither raises nor inhibits RNA synthesis. This suggests that, unlike dihydrotestosterone, 3$\beta$-androstanediol may have no function in the cellular maintenance of human BPH.

In human benign prostatic hyperplasia the conversion of testosterone to dihydrotestosterone occurs predominantly in the glandular components of the tumour (Siiteri and Wilson, 1970; Becker *et al.*, 1972; Harper *et al.*, 1974). This may imply that the stromal growth is not actively promoted by androgens and that it merely supports the maintenance of the epithelial elements. Our results show, however, that the smooth muscle which forms a substantial part of the stroma is hormone responsive and that RNA synthesis is influenced in a similar way as in epithelium.

Exposure to testosterone and dihydrotestosterone increases the uptake of $^3$H-uridine, while treatment with oestradiol reduces it. The finding suggests that the growth of the stroma, like that of the epithelium, may be controlled by steroid hormones and that this response is an additional and important factor to be considered in the evaluation of hormonal effects for clinical therapy.

We would like to thank Mrs Liesbeth Brown for skilled technical assistance and Mr Peter Lancaster for the preparation of the histograms and help with the microphotography.

The work was supported by the Cancer Research Campaign.

### REFERENCES

Baulieu, E. E., Lasnitzki, I. & Robel, P. (1968) Metabolism of Testosterone and Action of Metabolites on Prostate Glands Grown in Organ Culture. *Nature, Lond.*, **219**, 1155.

Becker, H., Kaufman, J., Klosterhalfen, H. & Voigt, K. H. (1972) *In vivo* Uptake and Metabolism of ($^3$H) testosterone and ($^3$H) dihydrotestosterone by Human Benign Prostatic Hypertrophy. *Acta endocr. Copenh.*, **71**, 589.

Brehmer, B., Marquardt, H. & Madsen, P. (1972) Growth and Hormonal Response of Cells Derived from Carcinoma and Hyperplasia of the Prostate in Monolayer Cell Culture. A Possible *in vitro* Model for Clinical Chemotherapy. *J. Urol.*, **108**, 890.

Bruchovsky, N. & Wilson, J. D. (1968) The Conversion of Testosterone to 5$\alpha$-androstan-17$\beta$-ol-3-one by Rat Prostate *in vivo* and *in vitro*. *J. biol. Chem.*, **243**, 2012.

Fraley, E. F., Ecker, S. & Vincent, M. (1970) Spontaneous *in vitro* Neoplastic Transformation of Adult Human Prostatic Epithelium. *Science, N.Y.*, **170**, 540.

Gittinger, J. W. & Lasnitzki, I. (1972) The Effect of Testosterone Metabolites on the Fine Structure of the Rat Prostate Gland in Organ Culture. *J. Endocr.*, **52**, 459.

Harper, M. E., Pike, A., Peeling, W. B. & Griffiths, K. (1974) Steroids of Adrenal Origin Metabolised by Human Prostatic Tissue both *in vivo* and *in vitro*. *J. Endocr.*, **60**, 117.

Houck, J. C. & Hennings, H. (1973) Chalones: Specific Endogenous Mitotic Inhibitors. *FEBS Letters*, **32**, 1.

Lasnitzki, I. (1970*a*) The Rat Prostate Gland in Organ Culture. In *Third Tenovus Workshop, Cardiff:* In some Aspects of the Aetiology and Biochemistry of Prostatic Cancer, Eds K. Griffiths and C. G. Pierrepoint. Cardiff: Tenovus Workshop Publications. p. 67.

Lasnitzki, I. (1970*b*) The Action of Testosterone and its Metabolites on the Rat Prostate Gland Grown in Organ Culture. In *Advances in the Study of the Prostate.* Eds M. H. Briggs and M. Stainford. London: William Heinemann Medical Books Ltd. p. 65.

Liao, S. & Fang, S. (1969) Receptor Proteins and the Mode of Action of Androgens on Gene Transcription in the Ventral Prostate. Review, *Vitamins and Hormones*, **27**, 17.

Morgan, J. F., Morton, H. J. & Parker, R. C. (1950) Nutrition of Animal Cells in Tissue Culture. *Proc. Soc. exp. Biol. Med.*, **73**, 1.

McMahon, M. J. & Thomas, G. H. (1973) Morphological Changes of Benign Prostatic Hyperplasia in Culture. *Br. J. Cancer*, **27**, 323.

McRae, C. U., Ghanadian, K., Fotherby, K. & Chisholm, G. D. (1973) The Effect of Testosterone on the Human Prostate in Organ Culture. *Br. J. Urol.*, **45**, 156.

New, D. A. T. (1966) *The Culture of Vertebrate Embryos.* London: Logus Press and Academic Press. p. 12.

Schrodt, G. R. & Foreman, C. D. (1971) *In vitro* Maintenance of Human Hyperplastic Prostatic Tissue. *Invest. Urol.*, **9**, 85.

Siiteri, P. K. & Wilson, J. D. (1970) Dihydrotestosterone in Prostatic Hypertrophy. 1. The Formation and Content of Dihydrotestosterone in the Hypertrophied Prostate of Man. *J. clin. Invest.*, **49**, 1737.

Trowell, O. A. (1959) The Culture of Mature Organs in a Synthetic Medium. *Expl cell. Res.*, **16**, 118.